# Development of Multimodal Fusion Technology for Tomato Maturity Assessment

**DOI:** 10.3390/s24082467

**Published:** 2024-04-11

**Authors:** Yang Liu, Chaojie Wei, Seung-Chul Yoon, Xinzhi Ni, Wei Wang, Yizhe Liu, Daren Wang, Xiaorong Wang, Xiaohuan Guo

**Affiliations:** 1 Beijing Key Laboratory of Optimization Design for Modern Agricultural Equipment, College of Engineering, China Agricultural University, Beijing 100083, China; 2 Quality & Safety Assessment Research Unit, U. S. National Poultry Research Center, USDA-ARS, 950 College Station Rd., Athens, GA 30605, USA; 3 Crop Genetics and Breeding Research Unit, United States Department of Agriculture Agricultural Research Service, 2747 Davis Road, Tifton, GA 31793, USA

**Keywords:** multimodal fusion, tomato maturity, deep learning, non-destructive testing

## Abstract

The maturity of fruits and vegetables such as tomatoes significantly impacts indicators of their quality, such as taste, nutritional value, and shelf life, making maturity determination vital in agricultural production and the food processing industry. Tomatoes mature from the inside out, leading to an uneven ripening process inside and outside, and these situations make it very challenging to judge their maturity with the help of a single modality. In this paper, we propose a deep learning-assisted multimodal data fusion technique combining color imaging, spectroscopy, and haptic sensing for the maturity assessment of tomatoes. The method uses feature fusion to integrate feature information from images, near-infrared spectra, and haptic modalities into a unified feature set and then classifies the maturity of tomatoes through deep learning. Each modality independently extracts features, capturing the tomatoes’ exterior color from color images, internal and surface spectral features linked to chemical compositions in the visible and near-infrared spectra (350 nm to 1100 nm), and physical firmness using haptic sensing. By combining preprocessed and extracted features from multiple modalities, data fusion creates a comprehensive representation of information from all three modalities using an eigenvector in an eigenspace suitable for tomato maturity assessment. Then, a fully connected neural network is constructed to process these fused data. This neural network model achieves 99.4% accuracy in tomato maturity classification, surpassing single-modal methods (color imaging: 94.2%; spectroscopy: 87.8%; haptics: 87.2%). For internal and external maturity unevenness, the classification accuracy reaches 94.4%, demonstrating effective results. A comparative analysis of performance between multimodal fusion and single-modal methods validates the stability and applicability of the multimodal fusion technique. These findings demonstrate the key benefits of multimodal fusion in terms of improving the accuracy of tomato ripening classification and provide a strong theoretical and practical basis for applying multimodal fusion technology to classify the quality and maturity of other fruits and vegetables. Utilizing deep learning (a fully connected neural network) for processing multimodal data provides a new and efficient non-destructive approach for the massive classification of agricultural and food products.

## 1. Introduction

In recent years, market globalization has significantly increased the demand for high-quality products, underscoring the necessity of rapidly, non-destructively evaluating produce maturity, such as predicting the ripening stages and storage shelf lives of fruits and vegetables like tomatoes [1]. The accurate assessment of maturity also plays a key role in supply chain management. Tomatoes, which are rich in vitamins A and C, boast bioactive components with antioxidant properties—carotenoids, flavonoids, and phenolic acids [2]. Furthermore, their antioxidant, anti-inflammatory, and antihypertensive properties have been linked to reducing the risk of cardiovascular diseases [3]. As tomatoes continue to ripen and undergo physiological changes post harvest [4], variability in their maturity post harvest can lead to higher spoilage risks in over-ripe tomatoes, resulting in significant losses during transportation and stores. Therefore, the objective and accurate classification of tomato maturity at harvest is crucial for grading and marketing, which could enhance the farm gate value of tomato production.

Traditional methods for assessing fruit and vegetable maturity, such as compression and puncture tests, have been widely used but are destructive and compromise the profitability of produce [5,6]. With technological advancements, a range of non-destructive techniques, including haptics [7,8], machine vision [9,10], visible and near-infrared spectroscopy (Vis/NIR) [11,12], hyperspectral imaging [13,14], electronic noses (e-noses) [15,16], and magnetic resonance imaging (MRI), now facilitate comprehensive maturity detection without damage [17,18]. These methods enable the evaluation of both internal and external fruit characteristics, offering insights into multiple quality parameters simultaneously. Specifically, non-destructive techniques like machine vision, Vis/NIR spectroscopy, and haptics have become pivotal in assessing tomato maturity and color grades. Machine vision, enhanced by deep learning, has shown remarkable success in classifying tomato maturity, with accuracies exceeding 90.7% [19,20,21]. However, while it is adept at identifying external maturity indicators, machine vision struggles with internal changes. Conversely, Vis/NIR spectroscopy has been effective in examining internal attributes, with various wavelengths revealing distinct ripening stages [22,23]. Additionally, the evolution of flexible sensor technology has propelled the use of tactile sensors in robotic applications, offering precise maturity assessments through firmness detection [24,25,26,27].

Assessing fruit maturity is a complex process encompassing color, internal quality, and firmness [28]. Current maturity detection often relies on a single non-destructive testing (NDT) method, yielding partial information that may lead to inaccurate maturity assessments. While imaging techniques can evaluate appearance, they often cannot adequately assess internal quality and firmness. Similarly, Vis/NIR spectroscopy offers insights into internal quality via spectral data, and tactile sensing technology captures fruit firmness through signal strength. However, each method’s perspective does not fully address the multidimensional nature of fruit maturity. Multimodal fusion technology, which integrates data from multiple NDT methods, has emerged as a solution to overcoming these limitations, enhancing accuracy and compensating for the limitations of individual methods. Currently, multimodal fusion techniques have been used in a variety of fields, such as medical diagnosis [29], emotion recognition [30], education [31], industrial fault diagnosis [32], and autonomous driving [33]. A number of reports have applied multimodal fusion technology to agriculture-related research [34,35,36,37,38]. The application of multimodal fusion technology in agricultural technology, especially in the assessment of fruit and vegetable maturity, not only improves the accuracy and efficiency of the assessment but also provides a new perspective for agricultural production and quality assessment supply chain management.

The utilization of deep learning for determining the maturity of fruits and vegetables has garnered significant interest lately. Researchers are increasingly applying deep learning algorithms to decipher ripening data for maturity assessments. For instance, Suharjito et al. [39] enhanced the quality of oil palm fruits by integrating deep learning with machine vision for maturity detection and classification. Similarly, Raghavendra et al. [40] leveraged a combination of convolutional neural networks (CNNs) and multilayer perceptrons (MLPs) with data from RGB and hyperspectral imaging to accurately identify banana maturity, achieving a remarkable accuracy of 98.4%. Deep learning not only increases recognition accuracy but also expedites processing, facilitates the extraction of features from intricate datasets, and enables precise classification and prediction.

This study aimed to classify tomato maturity through a tri-modal fusion approach incorporating imaging, Vis/NIR spectroscopy, and haptic technologies. Its significant contributions include the following: (1) establishing a multimodal tomato dataset reflecting various maturity stages, using data comprising RGB images, transmission spectra, and haptic signals, categorized into immature, semi-mature, and mature stages; (2) analyzing disparities in ripening within and on the surface of tomatoes that lead to misjudgments when using single-modal technologies and addressing these through multimodal fusion techniques; and (3) designing a multimodal fusion classification network for accurate maturity estimation.

## 2. Materials and Methods

### 2.1. Experimental Design

**Figure 1:**
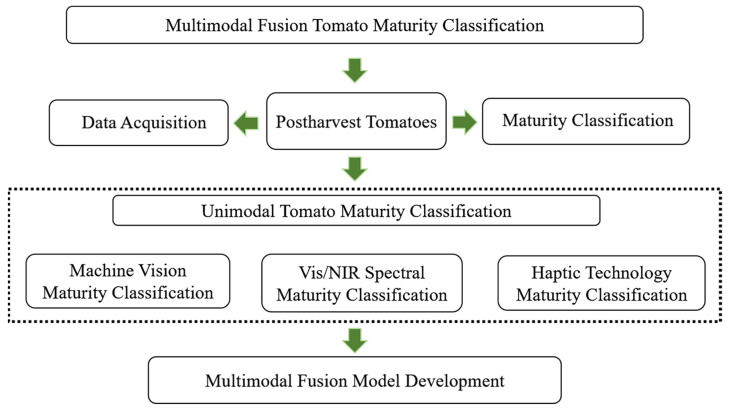
Flowchart of multimodal fusion tomato maturity prediction process.

Tomatoes without stems were obtained from a greenhouse farm in Tongzhou District, Beijing, China. Based on USDA standards [41] and previous studies [23,42], the color of the exocarp and cut surface along the equator of each tomato was obtained. The maturity categories in this study are defined as follows: immature (less than 10% red on the exocarp and cross-section), mature (more than 90% red on the exocarp and cross-section), and semi-mature (between 10 and 90% red on the exocarp or cross-section). In total, 214 tomatoes were classified into 79 immature, 60 semi-mature, and 75 mature tomatoes. In order to further study the multimodal technique, we also selected two negative samples and one positive sample for analysis. Negative sample 1 tomatoes had a red pericarp but a light green inner fruit cavity and flesh during the maturing process; negative sample 2 tomatoes had a red pericarp but a white inner cavity; and positive sample tomatoes had a uniform red color inside and outside for the best maturity. To mitigate environmental impact on prediction accuracy, the tomato samples were acclimated to a laboratory setting at 20 °C and 60% relative humidity for 12 h. To minimize error and ensure data reliability, each of the 214 tomato fruits was measured four times along the equatorial plane using each of the three devices, rotating 90° between acquisitions. This process generated 2568 data acquisitions encompassing image, spectral, and haptic modalities. Figure 1 shows a flowchart of the multimodal fusion tomato maturity prediction process.

### 2.2. Data Acquisition

#### 2.2.1. Image Acquisition

**Figure 2:**
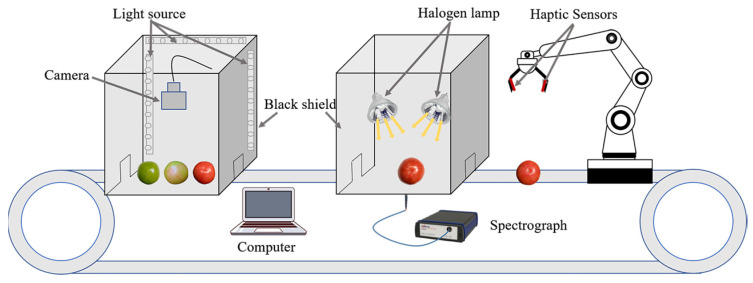
Diagram of data acquisition device.

A schematic diagram of the data acquisition device, as shown in Figure 2, shows the acquisition devices for image, spectral, and tactile information from left to right. 

Color images were captured using a DaHeng MER-030-120UC color industrial camera. The tomatoes were placed on a conveyor platform and rolled along it. Each tomato fully entered the camera’s field of view for image acquisition. It was rotated 90° for each acquisition and turned four times to capture a complete image of the tomato’s surface. The surfaces of the light sources were equipped with diffuse reflectors to prevent any exposure on the tomato’s outer surface. Each diffuse reflector was located between the light source and the sample and was made of frosted glass.

#### 2.2.2. Vis/NIR Spectral Information Acquisition

Spectral data were acquired using an AvaSpec-ULS2048XL-EVO spectrometer, covering a wavelength range of 350 nm to 1100 nm. Two 250 W halogen lamps, positioned 20 cm away from the sample, served as light sources in a transmission-based acquisition system. To guarantee the light’s stability and uniformity, the lamps were preheated for 15 min prior to the experiments. The sample was positioned on a fruit cup, beneath which optical fibers were arranged to capture spectral information. Data acquisition was facilitated through an external trigger mechanism employing a photoelectric switch to gather spectral data. The integration time was set to 35 ms, with averaging performed five times to ensure accuracy and consistency in data collection.

#### 2.2.3. Tactile Information Acquisition

The haptic information acquisition system utilized flexible thin-film pressure sensors (i-Motion, model IMS-C10A) attached to both sides of mechanical jaws to detect the sample’s firmness. Data collection was conducted at a sampling frequency of 100 Hz for 15 s. The method entailed positioning a tomato on the experimental platform, maneuvering the robotic arm toward the sample at a set speed of 0.5 m/s, and then clamping the mechanical jaws to secure the tomato and collect data. To mitigate the impact of variable surface firmness on tomatoes, data were obtained from four equatorial points on each tomato. The overall pressure value for each tomato was calculated by averaging these four readings.

### 2.3. Data Preprocessing

Corresponding preprocessing was performed for the image, spectral, and tactile raw data, respectively, to reduce noise and improve the signal-to-noise ratio.

The chromatic distinction between the tomato and its background was pronounced. For image processing, we converted the image to grayscale and applied a fixed threshold of 50 to separate the tomato from the background. Subsequently, the pixel dimensions of the segmented image were standardized to 150 × 150.

Spectral data underwent preprocessing to counteract noise and bias from the spectrometer’s dark current, including a black-and-white correction based on reference data obtained with the light source on (*R_w_*) and off (*R_d_*), using the formula

(1) $$R=\frac{R_{raw}-R_{d}}{R_{w}-R_{d}}\;$$

The haptic data experienced instability during collection which was attributed to mechanical vibrations. We designed a Butterworth low-pass filter to eliminate the impact of environmental factors. We set the cutoff frequency of the filter to 10 Hz and the order to 4 for filtering.

### 2.4. Sample Quality Measurement

The firmness of the fruit was defined as the force (F) per unit area (A) using the equation P = F/A, and it was computed using the measured force value in newtons and the probe’s contact area in square meters, where P was the firmness of the measured fruit and the unit was Pa. The firmness of the tomato fruit was measured by pushing the plunger tip (8 mm) into the opposite cut surface along the equatorial region using a hand-held penetrometer (GY-4, HANDPI, Beijing, China), and the value was expressed in Pa. Subsequently, the SSC of each tomato fruit was determined using traditional destructive methods. Each tomato was cut into small pieces, juice was then extracted from the entire tomato and filtered using double-layer gauze, and 1 mL of juice was dropped onto a fruit sugar refractometer (PAL-1, ATAGO, Tokyo, Japan).

### 2.5. A Deep Learning Framework for Multimodal Fusion

Multimodal fusion involves combining data from different modalities. Commonly used multimodal information fusion methods include feature-level fusion, decision-level fusion, and model-level fusion [43]. This study employed a multimodal feature fusion scheme. Following data collection, we performed data preprocessing and feature extraction for all modalities, including background removal for images, a black-and-white correction for spectral data, and filtering for tactile data. The features were then fused, and the fused features were input into a fully connected neural network for maturity prediction, as described next.

#### 2.5.1. Feature Extraction

**Figure 3:**
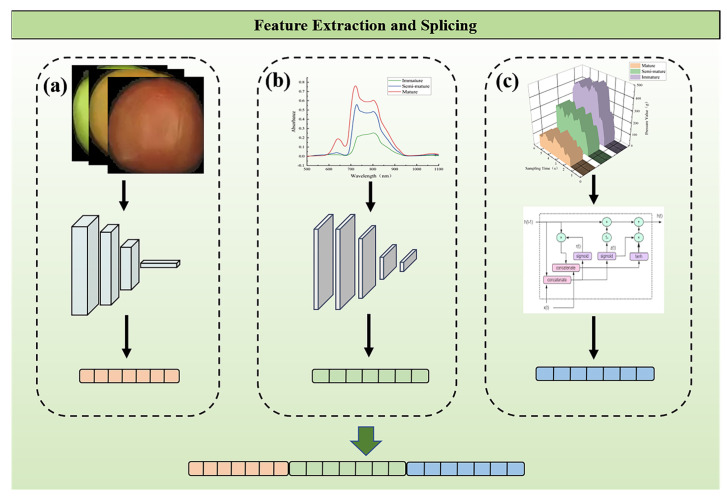
Diagram of feature extraction and fusion. (**a**) Image feature extraction; (**b**) Vis/NIR spectral feature extraction; (**c**) haptic feature extraction.

Visual Geometry Group 16 (VGG16), developed by Oxford University’s Visual Geometry Group in 2014, is a convolutional neural network adept at processing image data [44]. VGG16 consists of five convolutional blocks and three fully connected layers. The first two convolutional blocks are composed of two convolutional layers and one pooling layer, and the last three convolutional blocks are composed of three convolutional layers and one pooling layer. The process of image feature extraction is shown schematically in Figure 3a. This study utilized VGG16 for tomato image feature extraction, as depicted in Figure 3a. By inputting pre-processed tomato images into the VGG16 model, we extracted a 1 × 8192-dimensional feature vector from the output of the model’s fully connected layer, representing the tomato image feature.

The primary benefit of CNN lies in its algorithmic strategy for directly extracting features from raw input data [45]. Its capabilities in learning and classification surpass those of conventional neural networks. Hence, a 1D-CNN model was developed for spectral feature extraction, as depicted in Figure 3b. The 1D-CNN model was composed of three convolutional layers; each layer was followed by batch normalization, a ReLU activation function, and a maximum pooling layer, and, finally, the classification results were output through two linear layers. The de-noised spectral data were fed into the 1D-CNN model, in which the convolution layer served the purpose of feature extraction, yielding a 1 × 10-dimensional spectral feature vector.

A Long Short-Term Memory (LSTM) network, a specialized variant of Recurrent Neural Network (RNN), incorporates long-term memory into an RNN by facilitating constant error backpropagation through its internal memory cells [46]. This attribute renders it exceptionally suitable for processing sequences with significant temporal structures, finding extensive application in tactile information processing [47,48]. An LSTM network was designed to decode one-dimensional tactile signals, as depicted in Figure 3c, and comprised three gates (input, forget, and output) and a unit state. This structure allowed the network to retain information over ambiguous time periods and to learn detailed temporal features. Our haptic dataset was input into the model, and the model fully learned the tactile feature information and finally extracted a 1 × 64-dimensional tactile feature vector to represent the texture information of tomato.

#### 2.5.2. Feature Fusion

Various feature fusion techniques exist, such as feature weighting [49] and feature mapping [50], yet these approaches often necessitate complex model adjustments and might lead to information loss. Feature weighting may fail to fully capture information across all modes, and feature mapping could compress information, omitting vital details. In multimodal feature fusion, early data fusion occurs at the input layer, known as feature stitching [37]. We utilized feature splicing to merge image, spectral, and haptic data in the fusion process. This method combined different modes into a single multimodal input, [m, n, z], where m, n, and z denote the feature vectors for image, spectral, and haptic data, respectively. Thus, we created a comprehensive cross-modal feature vector with a dimensionality of 1 and a total length of 8266, equal to the sum of the three feature vectors. These spliced feature vectors were then input into the deep learning model for training, validation, and testing.

This approach not only augmented feature dimensionality but also preserved the integrity of each data type. By enabling the deep learning network to independently assess the relevance of each data type, we enhanced information richness and reduced error potential. This method offered a nuanced and thorough analysis, allowing for comprehensive insights into tomato maturity.

#### 2.5.3. Multimodal Fusion Classification Networks

**Figure 4:**
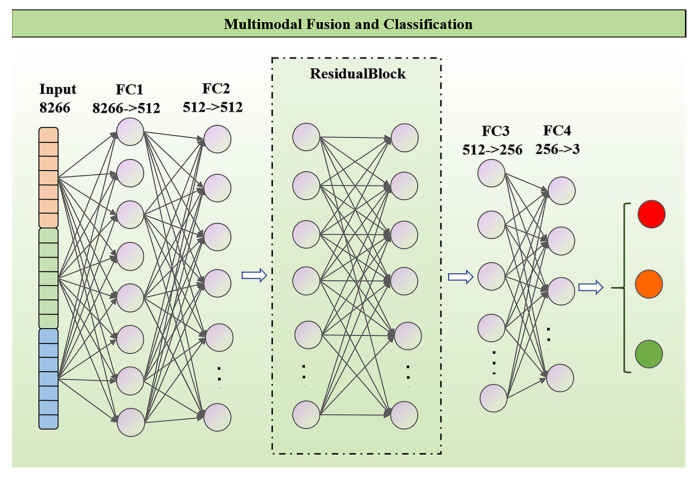
Multimodal fusion fully connected network structure.

In this study, a fully connected deep neural network model was proposed for processing multimodal data, as shown in Figure 4. The model adopted a multi-layer fully connected architecture that incorporated a residual learning mechanism to enhance the model’s learning ability and generalization performance when processing complex datasets. The model had a total of four fully connected layers, each of which contained 8266, 512, 512, and 256 neurons, respectively, and each fully connected layer was immediately followed by a batch normalization layer, a ReLU activation function, and a Dropout layer. The first two fully connected layers were used for feature extraction, and two fully connected layers were used for classification. In addition, a residual block was introduced between the second and third fully connected layers which contained two fully-connected layers, and the two connected layers were followed by the batch normalization and Dropout layers after each layer. The batch normalization layer was used to normalize the output of the layer to improve training stability and efficiency. The ReLU activation function was applied to introduce nonlinearities and enhance the expressive power of the model. The Dropout layer prevented overfitting by setting a reasonable scale size. The whole network realized the efficient processing of multimodal data through this layered structure and residual learning strategy.

### 2.6. Model Evaluation

To comprehensively evaluate the model performance, we chose three key metrics for the classification model, namely, accuracy, precision, and recall, to evaluate the model.

A TP (True Positive) result indicated that the actual situation was a positive situation and the prediction was positive, i.e., the prediction was correct, and the same result could be obtained for TN (True Negative), FP (False Positive), and FN (False Negative) results [51].

Accuracy reflected the proportion of samples that the model recognized correctly and was a fundamental measure of the overall performance of the model.

(2) $$Accuracy=\frac{TP+TN}{TP+TN+FP+FN}$$

The precision rate, on the other hand, focused on the proportion of samples predicted by the model to be positively classified over all positively classified samples (TP + FP) and focused on assessing the classification accuracy of the model.

(3) $$Precision=\frac{TP}{TP+FP}$$

Recall measured the proportion of positively classified samples identified by the model among all positively classified samples (TP + FN), and it is critical for assessing the model’s ability to capture positively classified samples.

(4) $$Recall=\frac{TP}{TP+FN}$$

These three indicators together constitute a comprehensive and in-depth system of evaluating the model’s performance which can reflect the performance of the model in multimodal data processing.

The experimental computer platform utilized Windows 10 Professional 64-bit on an Intel Core i7-7700HQ @ 2.80GHz quad-core processor. The maturity assessment model was developed using PyCharm IDE and the PyTorch framework, with training conducted on an NVIDIA GeForce GTX 1050 Ti 4GB GPU.

## 3. Results and Discussion

### 3.1. Analysis of Soluble Solids and Firmness of Tomatoes

**Figure 5:**
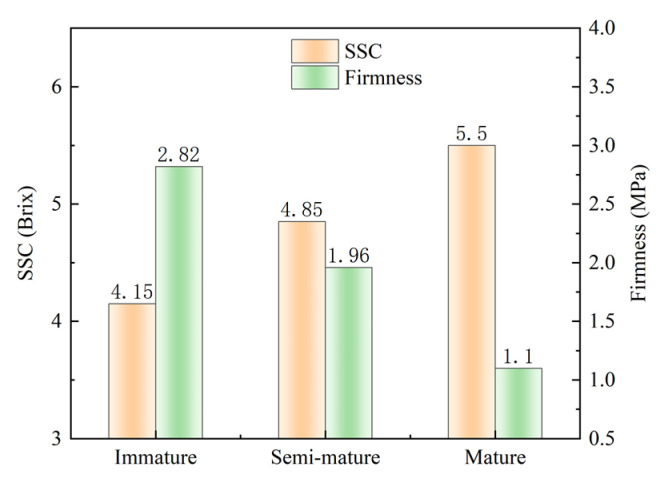
Soluble solids content and firmness values of tomatoes in different maturity stages.

The internal nutrients of tomatoes change during ripening in addition to changes in the exocarp and internal color. This study also investigated changes in the soluble solids content and hardness during the tomato ripening process, which can further be used for the establishment of SSC and hardness prediction models in future research. As shown in Figure 5, the soluble solids content of the tomatoes showed an ascending trend as they ripened, while the firmness of the tomatoes decreased gradually. The mean SSC values of tomatoes in different ripening periods were 4.15, 4.85, and 5.5 Brix, and the mean values of firmness were 2.82, 1.96, and 1.1 MPa. These findings indicate a clear negative correlation between the soluble solids content and fruit firmness during the tomato maturation process. However, these traditional methods of maturity assessment are destructive, posing challenges for rapid and non-destructive testing requirements in fruit and vegetable quality monitoring and inspection.

### 3.2. Analysis of Original Data

#### 3.2.1. Image Data Analysis

**Figure 6:**
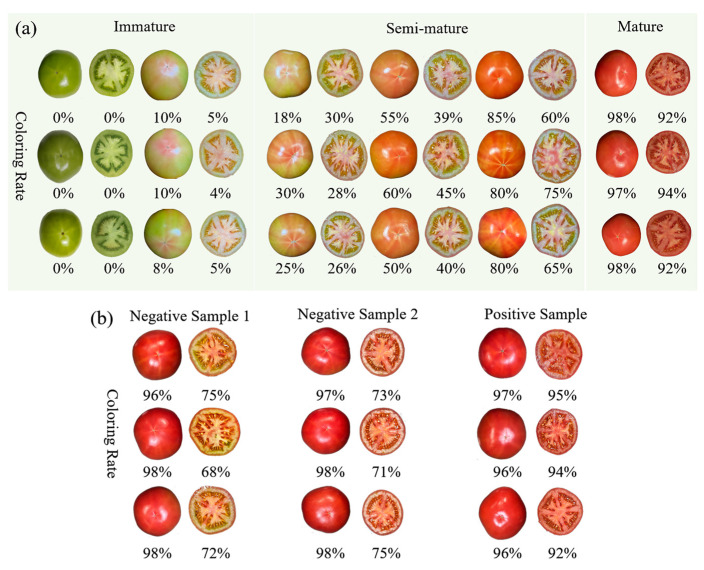
Images of tomatoes at different ripening stages; (**a**) images of tomatoes at three ripening stages: immature, semi-mature and mature; and (**b**) images of cross-sectioned unevenly mature tomatoes.

As illustrated in Figure 6a, significant color changes were observed in the tomatoes during ripening. The exocarp and mesocarp evolved from greenish-white to pink and finally to bright red, while the endocarp transitioned from green to white to red. Similarly, the funiculus color shifted from green to light yellow, culminating in red at full maturity. These color changes effectively illustrate the progression from immature to semi-mature and ultimately mature stages, highlighting the dynamic nature of the ripening process. Images, as the most commonly used means of determining tomato ripeness, are capable of capturing color changes in exterior appearance, and, as shown in Figure 6a, image processing techniques are able to calculate the percentage of red color on the surface of a tomato and classify it correctly, thus accurately determining tomato ripeness by appearance. Due to a variety of factors, tomato maturity was not uniform, and there were cases in which the exterior was mature but the interior was not, making it impossible to identify the interior color and texture from the exterior image. As shown in Figure 6b, we analyzed three categories of tomatoes with a red appearance but different internal maturity conditions and determined by their appearance that the tomatoes had all reached maturity. Negative samples 1 and 2 were judged to be mature according to their appearance; the outer skin color ratio reached more than 95% but the interior was not mature, and the color ratio was about 70%. The color ratio of the positive sample reached more than 95%, showing the best mature state. Immature tomatoes contain lycopene, which can be harmful to the human body after consumption [52], so it is necessary to combine a variety of nondestructive testing techniques to determine the maturity of tomatoes comprehensively.

#### 3.2.2. Analysis of Spectral Data

**Figure 7:**
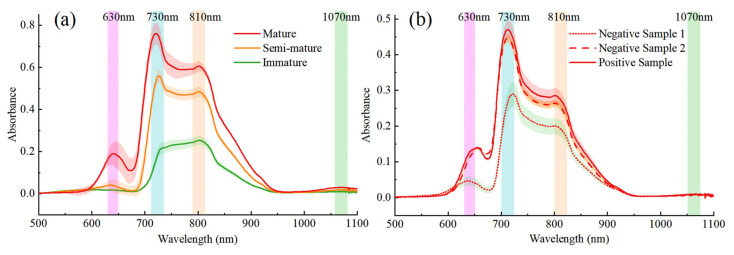
Spectral information of tomatoes at different maturity stages; (**a**) spectral information of tomatoes at three ripening stages: immature, semi-mature, and mature; (**b**) spectral information of cross-sectioned unevenly mature tomatoes.

The ripening of the tomatoes was marked by significant changes in their internal nutrient composition, including a decrease in chlorophyll content and an increase in anthocyanin content [53]. These alterations are mirrored in the measured spectral absorbance curves presented in Figure 7a. The curves demonstrate consistent trends across different maturity stages, with mature tomatoes exhibiting higher spectral intensities between 600 and 950 nm. Notable absorption peaks were observed at 630 nm, 730 nm, 830 nm, and 1070 nm, corresponding to chlorophyll pigments, the O-H band’s third overtone, the C-H band’s fourth overtone, and the absorption region for carbohydrates [54]. Additionally, the 970 nm–1180 nm range was influenced by O-H bonds [22]. During tomato ripening, a decrease in chlorophyll content and an increase in lycopene combined to cause a change in the light transmission spectral curve of the tomato. These changes indicate a significant correlation between the optical properties of tomatoes and their natural growth processes. The spectra also can discriminate between unevenly internally and externally ripened tomatoes. The mean spectra with standard deviation values for the three categories of tomatoes, namely negative sample 1, negative sample 2, and the positive sample, are depicted in the Figure 7b. The spectral trends of the three types of samples are basically consistent. There is a slight difference in spectral signals between negative sample 2 and the normal sample, but they have a significant difference compared to negative sample 1. For similar maturity, Vis/NIR spectroscopy could not completely differentiate maturity categories, so it was necessary to further rely on other NDT measurements. In daily life, touching tomatoes with hands to determine their texture is one of the important ways of judging ripeness in addition to vision. Therefore, haptic technology was introduced to solve the problem of tomato ripeness assessment.

#### 3.2.3. Analysis of Haptic Data

**Figure 8:**
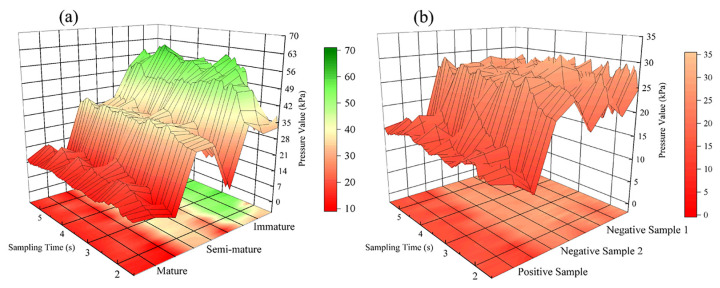
The haptic information of tomatoes at different maturity stages; (**a**) the haptic information of tomatoes at three maturity stages: immature, semi-mature, and mature; (**b**) the haptic information of cross-sectioned unevenly mature tomatoes.

Pronounced changes in tomato firmness throughout ripening were depicted using 3D color-mapped surfaces of tactile pressure signals (Figure 8a). Mature tomatoes exhibited pressure values ranging from 13 to 26 kPa, semi-mature tomatoes exhibited pressure values ranging from 26 to 46 kPa, and immature tomatoes exhibited pressure values ranging from 52 to 65 kPa. These variations were attributed to the enzymatic breakdown of pectin, leading to tissue softening [55]. Consequently, the marked differences in firmness at each ripening stage suggest a potential for non-destructive maturity assessments through haptic analysis, offering a straightforward method for evaluating tomato quality. In a case of heterogeneous internal and external ripening, the interior of negative sample 2 was off-white and the texture of the tomato was hard, so we utilized the characteristic of negative sample 2’s hard texture to differentiate negative sample 2 from the positive sample by the haptic technique. As shown in Figure 8b, negative samples 1 and 2 were less mature and had a hard texture, but the mature positive sample had a softer texture. From the data, the pressure values of negative samples 1 and 2 reached approximately 29 kPa, and the pressure value of the positive sample reached about 13 kPa. Therefore, negative sample 2 and the positive sample could be distinguished through the difference in pressure values, thereby making up for the inability of the spectrum to distinguish samples from the two groups.

Although the internal ripening situation could not be identified through external color in the images, the combination of near-infrared spectroscopy and haptic sensing technology avoided the shortcomings of the images. The study results suggest that during the ripening process of tomatoes, the apparent color, internal structure, and firmness of tomatoes became inhomogeneous, resulting in inaccurate single-modal maturity classification, and the interference of other factors during the ripening process could not be ruled out.

### 3.3. Multimodal Fusion Maturity Classification Model

#### 3.3.1. Unimodal Maturity Classification

Before performing multimodal fusion classification, we first built unimodal deep learning classification models for the three categories of tomato maturity: immature, semi-mature, and mature. We developed three deep learning models for this purpose: a VGG16 model for imaging, a one-dimensional CNN for spectral analysis, and an LSTM network for haptic data. The dataset was split as follows: 64% for training, 16% for validation, and 20% for testing. The dataset was divided into a training set, test set, and validation set at a ratio of 8:4:5. The outcomes of model training are detailed in Table 1.

Table 1 shows the accuracy, precision, and recall of the models built on images, spectra, and haptics, and the performance of the test set is further discussed. The three optimal image, spectral, and haptic unimodal classification models reached 99.2%, 87.8%, and 87.2% accuracy and 89.9%, 89.9%, and 99.2% precision. The recall rates of the three optimal unimodal classification models showed poor levels in general, especially for the spectral and haptic classification models that demonstrated only 64.8% and 66.7%, respectively. VGG16 demonstrated high accuracy in classifying tomato maturity, proving its effectiveness in distinguishing image variations associated with maturity levels. This underscored the potent utility of images in discerning tomato ripeness. However, images fell short in detecting the internal ripening stages of tomatoes, necessitating the integration of additional methods for a thorough analysis of tomato maturity. The spectral and haptic analyses showed lower accuracy values, particularly in recall rates. This could be attributed to the models’ inability to adequately capture features essential for differentiating between categories, complicating the accurate identification of specific sample categories during prediction. Consequently, for spectral and tactile data, the deployment of more sophisticated models or enhanced feature engineering might be necessary to unearth more distinctive characteristics. For instance, employing more advanced deep learning frameworks or developing custom models tailored to specific tasks could have enhanced model accuracy.

#### 3.3.2. Multimodal Fusion Maturity Classification

**Figure 9:**
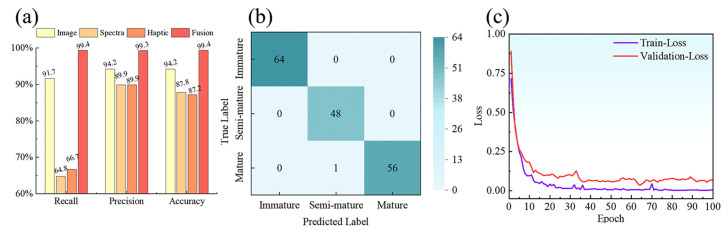
Comparison of model (**a**) evaluation metrics, (**b**) confusion matrix, and (**c**) Loss.

To evaluate the efficacy of the multimodal fusion technique, we conducted a comparative analysis between unimodal and multimodal fully connected neural network (FCNN) classification models across image (VGG16), spectral (1D-CNN), and haptic (LSTM) modalities, focusing on recall, precision, and accuracy metrics. Figure 9a showed that the FCNN model achieved the highest performance, with 99.4% recall, 99.3% precision, and 99.4% accuracy. In comparison, the VGG16 model showed 91.7% recall and over 94% for both precision and accuracy. The 1D-CNN and LSTM models demonstrated lower recall rates (66.7% and 64.8%, respectively) but maintained high precision at 89.9%, with accuracy rates of 87.2% and 87.8%. Overall, the FCNN model shows good comprehensive performance, and the recall, precision and accuracy in the test set of the FCNN model are significantly improved compared to the unimodal classification model, with an up to 34.6% improvement in recall and up to 9.4% and 12.2% improvements in precision and accuracy. The effectiveness and superiority of multimodal fusion methods in deep learning classification tasks are demonstrated.

To examine the multimodal fusion classification network from multiple perspectives and demonstrate the classification results more intuitively, we plotted a confusion matrix of the FCNN model’s classification results in Figure 9b. Only one mature sample is misclassified as a semi-mature sample, which may have been caused by the closer proximity of the test sample to the semi-mature sample, but overall, the overall classification accuracy is still high. In addition, the loss values (Loss) during the model training process used to comprehensively assess the overall ability of the model were examined, as shown in Figure 9c. The curves of the loss function values of the model on the training and test sets showed a fast decreasing trend and then gradually stabilized. After approximately 30 instances of training time, the loss values of the model’s training and test sets were stabilized at 0.03 and 0.09. The convergence of the model could be obtained through the Loss curve, which indicates that the model’s training effect is relatively good and the model was able to correctly capture the laws in the training data. By comprehensively analyzing the confusion matrix and loss curves, we were able to understand the classification performance, training process, and generalization ability of each model more comprehensively, thus providing a more solid basis for model selection and adjustment.

#### 3.3.3. Independent Validation of Heterogeneous Samples of Internal and External Maturity

**Figure 10:**
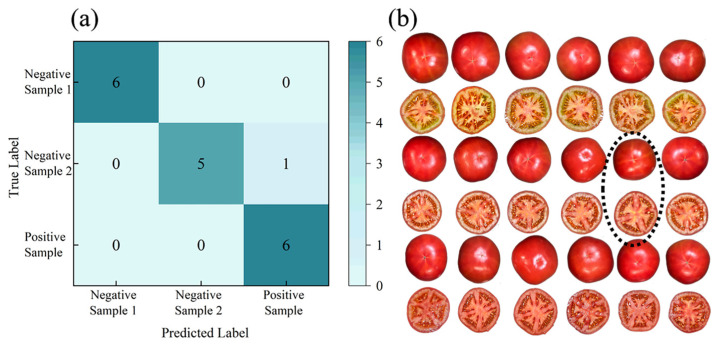
Validation results: (**a**) confusion matrix; (**b**) validation sample plot.

Beyond classifying tomatoes into immature, semi-mature, and mature categories based on apparent changes, the current study also extended to classifying tomatoes with uniform external redness but varied internal ripeness levels. We employed VGG16, 1D-CNN, and LSTM models to integrate the image, spectral, and haptic data into the fusion model for this purpose. A validation set of eighteen samples yielded an accuracy rate of 94.4%. The confusion matrix displayed in Figure 10a reveals a single instance in which a negative sample 2 sample was incorrectly classified as a positive sample. The misclassified sample is shown in Figure 10b. We measured a firmness value of 1.3 MPa for this sample. This could be due to the low firmness value of this sample, which resulted in the misclassification of this sample due to the close proximity of the three features extracted from the image, spectral, and haptic data to the positive sample.

## 4. Conclusions

In this research, a multimodal fusion approach was proposed for classifying the ripeness of tomatoes. Image, Vis/NIR spectral, and haptic data were collected and analyzed. For a comparison with the multimodal fusion approach, three single-modal models were developed, achieving an optimal performance of 94.2%. Further, a multimodal fusion model utilizing a fully connected neural network was established which attained the best classification accuracy of 99.4%, with a 5.2% improvement. The multimodal fusion network outperformed the three traditional single-modal models by achieving a 99.4% accuracy, 99.3% precision, and 99.4% recall in classifying tomato maturity. In particular, the classification accuracy reached 94.4% in the case of inconsistent internal and external maturity. These findings prove that multimodal fusion technology can overcome the limitations of single-modal classifications and highlights its specific advantages. The multimodal fusion method identified different ripened tomatoes, improved food quality, developed a new way of conducting rapid and non-invasive tomato maturity evaluations, and laid a foundation for optimizing harvest time and maximizing the value of tomato products.

Despite the remarkable results of this research obtained, there are still some challenges in integrating this technology into existing online sorting systems, such as the real-time responsiveness of data processing and sorting, the adaptability of the fusion technology to multiple fruits and vegetables, and the cost-effectiveness of sorting systems. Future research requires the optimization of algorithms and hardware design, incorporating more NDT techniques to explore the effectiveness of the large-scale online sorting of tomato ripeness and other quality control parameters, such as pests or virus-infected tomatoes. The multimodal fusion method can be used as a reference for other types of fruits and vegetables in the future, such as kiwi, persimmon, and cucumber. If widely used, this method can improve the quality of fruit and vegetable sales according to the different maturity stages of fruits and vegetables in graded sales, bring significant economic benefits, and improve consumer satisfaction. Therefore, multimodal fusion technology will have a wide impact on the development of agriculture and food quality control technology.

## Figures and Tables

**Table 1:** Tomato unimodal classification performance.

Models	Accuracy			Precision			Recall		
	Training Set	Validation Set	Test Set	Training Set	Validation Set	Test Set	Training Set	Validation Set	Test Set
Imagery	94.0%	93..4%	94.2%	94.5%	94.6%	94.2%	94.8%	94.7%	91.7%
Spectral	87.3%	84.7%	87.8%	89.7%	87.6%	89.9%	66.3%	65.2%	64.8%
Haptic	90.0%	88.7%	87.2%	90.4%	89.7%	89.9%	66.7%	66.7%	66.7%

## Data Availability

Data are contained within the article.
